# Supplementary material for: Analysis of factors impacting electoral integrity in Africa between 2006–2023 - Examining the association between free and fair election and rule of law
Source: PLoS One. 2025 Oct 9;20(10):e0334505. doi: 10.1371/journal.pone.0334505 (PMC12510509; doi:10.1371/journal.pone.0334505)
Supplement: Attachment — Submitted filename: Plos_One_Review.pdf [file pone.0334505.s001.pdf]

Thank you for the opportunity to review this manuscript. The paper addresses an important and timely topic by exploring potential factors that contribute to free and fair elections—specifically, rule of law, electoral pluralism, and political rights. Against the backdrop of limited democratization in many African countries despite the regular holding of elections, the authors seek to better understand which factors help foster elections that genuinely support democratic development.

The research question is highly relevant, and the dataset is well-suited to address it. However, I believe the methodological presentation and interpretation of results would benefit from restructuring to improve overall clarity. I recommend that the authors be given the opportunity to revise and resubmit the paper.

Currently, the empirical section is difficult to follow, particularly for readers who may not be familiar with advanced econometric techniques such as panel quantile regression. Although the models are introduced sequentially, it is not always clear how they relate to one another or why specific methodological choices—such as the use of quantile regression over other approaches—were made. I was also unsure why panel OLS regression appears as a robustness check when earlier models using OLS with one- and two-way fixed effects are presented as main models. Clarifying which models are considered the primary analyses and which serve as robustness checks, and explaining the rationale behind these choices, would certiainly help strengthen the empirical contribution.

Additionally, more detail is needed regarding the group referred to as the "twenty top democratizing countries" in Africa. How were these countries selected? Which data source (e.g., V-Dem?) was used to define this group? Why was the sample restricted to 20 countries? Providing this information would help readers better understand the case selection and its relevance to the broader argument.

Minor comments:

- The manuscript would benefit from careful proofreading and editing for grammar, clarity, and consistency of voice (e.g., switching between “we” and “I”).
- There is some repetition in the discussion of data sources, particularly across Sections 3.1 and 3.2, which can make the argument harder to follow.
- Ensure that all cited works appear in the reference list—for example, Edgeli (2018) is mentioned in the text but not included in the references.
