# Supplementary material for: Analysis of factors impacting electoral integrity in Africa between 2006–2023 - Examining the association between free and fair election and rule of law
Source: PLoS One. 2025 Oct 9;20(10):e0334505. doi: 10.1371/journal.pone.0334505 (PMC12510509; doi:10.1371/journal.pone.0334505)
Supplement: Attachment — Submitted filename: Response to Reviewer- Mike and Dilek.docx [file pone.0334505.s003.docx]

| **Reviewers’ Comment** | **Authors’ Responses** | |
| --- | --- | --- |
| **Reviewer #1:**  First, there should be a more **rigorous discussion of how each variable is measured using V-Dem** data to ensure that the rule of law variable is distinct from other confounders. | Thank you for your comment  A rigorous discussion on how each variables is mesaured to reflect how distinct they are usnig in V-Dem is presented from lines 242-254. The shows that every variable is distinct and not a duplicate or repetition.  “ To ensure that the rule of law was distinct from potential confounding variables, this study compared subdimensions of each variable as defined by the Varieties of Democracy (V-Dem) dataset. The **rule of law** subdimensions—judicial independence, compliance with judiciary decisions, and respect for legal procedures—primarily concern institutional judicial processes and enforcement. This creates a clear functional distinction from the subdimensions of **political rights,** which emphasize citizens' behavioral and participatory rights in the political process, such as freedom of association, freedom of expression, suffrage, and inclusive participation.  Similarly, **pluralism** is characterized by party-level engagement rather than individual political activities. Its subdimensions—participation of multiple political parties, the existence of opposition parties, and electoral competitiveness—address democratic variations from a supply-side perspective, distinct from the institutional, judiciary-centered nature of the rule of law. Lastly**, free and fair elections** specifically relates to electoral performance, encompassing subdimensions like electoral fraud, electoral violence, and voter registration integrity, thereby differing from the judicial performance captured within the rule of law dimension. ” | |
| Second, the author(s) **should justify their case selection**. Why do they limit analysis to the top 20 democratizers, and ho might this affect their findings? | Thank you for your comments.  According to Gerring (2007), in analyzing cases to provide more insight, non-random selection procedures that focus on most similar cases- in this article, the most democratizers – provide insight into factors promoting electoral integrity. They have been selected from VDEM 2024 data and examined for further understanding of democratization in Africa.  The top 20 democratizers represent a subset of African countries that have conducted regular elections and attained a certain level of integrity that improves democracy.  Hence, selection is justified by the performance of the group. 20 represents 40 per cent of African countries, which is a good representation.  These discussion has been provided from lines 268 – 280. | |
| **Reviewer #2:** |  |  |
| The abstract: The conclusion of the abstract is too theoretical. **We can do without that.** | Thank you for your comments  The statement “In contrast, nations with high levels of electoral integrity should consolidate and sustain established mechanisms.” has been removed. | |
| Line 98. We should have a footnote to indicate the figures that support this sentence. | The Economist Intelligence Unit and Freedom House statistics figures reflect the resurgence of coups in West Africa amid already existing autocracies. The referencing has been added. | |
| What is the contribution of this article to academic literature? | The below statement have been inserted in lines 187 - 198 to explain the significant contribution of this study to literature.  Discussion on protecting electoral integrity in emerging democracies, focusing on political institutions and processes. Recent studies examined the viability of using photo quick count to improve electoral integrity which argued that using photos and voice as auditory measures upholds electoral integrity; however, electoral pathologies that undermine integrity persist (20,21).Additional studies presents that that there is an increase in the legitimacy of electoral processes in emerging democracies, and fairness must move beyond the ballot. The analysis of electoral integrity perception in 18 countries from 2004 to 2021 was examined (23, 24).  Most of these studies examined emerging democracy while this study focused on the most performing African countries as emerging democracies. While other studies examined factors such as auditory measures within countries by focusing on impacting certifying elections by citizens and on electoral integrity perception, this study contributes to reexamining direct factors that impact electoral integrity rather than citizens satisfaction or perception in 20 countries. | |
| The author doesn't have a single way to cite references in the document's body. | Thanks for your observation  We have consistently use numbering pattern and it is consistent across the study.  We have removed the hanging footnote and two intext citations replaced with numbering to ensure consistency with the journal style. | |
| In the countries that make up your sample, **we don't find the countries cited in the introduction.** Why | Thank you for your comments.  The countries that make up the samples have been listed in the introduction; however, references have been made to only a few who have held elections recently and have experienced the most remarkable shift in the outcome of elections.  The below statemen have been inserted in lines lines 73 to 82.  “Using levels of electoral fraud, electoral violence, and voter registration integrity, V-Dem (2024) presents Nigeria, Ghana, South Africa, Egypt, Kenya, Benin, Botswana, Cape Verde, Cote d'Ivoire, Gambia, Lesotho, Liberia, Madagascar, Malawi, Mauritius, Namibia, Senegal, Tanzania, Uganda, and Zambia as top democratizing countries in Africa. The cases of South Africa, Nigeria, Botswana, and Cape Verde present successive elections and increasing democratization. The Liberian 2023 election reflects the citizens' will and significant legitimacy given the public opinion that certifies the result and welcomed President Joseph Boakai as opposed to former President George Weah. Similarly, the 2024 election in Botswana that ended the rule of a party that had governed the country since independence reflects increasing democratization in governance signalling remarkable openness of political space.”  Subsequently, other cases of democratic backsliding were mentioned to establish the mix of emerging democracies and autocracies in Africa as a problem. | |
| Abbreviations that are not explained previously. Example: CSDA, OLS, | These abbreviations have been identified and explained in the analysis sections indicated below.  Autoregressive Distributed Lag (ARDL)  **Cross-sectional dependency test (CSDT/CDSA)**  **Ordinary Least Square (OLS)**  Lines 245 – 246  Autoregressive Distributed Lag (ARDL), which analyses short-run and long-run relationships among variables regardless of stationary issues, has been employed to examine the long-run effect.  Lines 277 -280  **The Cross-sectional dependency test for examining cross-sectional units' interdependency has been employed for the detection of cross-sectional dependence and is analyzed through Pesaran's CD technique framework,**  **Lines 311 - 314**  To investigate the connection between free and fair elections as the dependent variable and rule of law, electoral pluralism, and political rights, this study used Ordinary Least Squares (OLS). This approach estimates the linear relationship among variables by minimizing the sum of the squared residuals. | |
| The results are not convincing. First, I don't understand the choice of Panel Quantile Regression. The author should provide reasons. | Thank you for this suggestion.  The results are convincing and the study employed Panel Quantile Regression as it allows exploring how the rule of law influences electoral integrity not just on average but across the full distribution of integrity scores—capturing whether its impact differs in countries with low versus high levels of free and fair elections.  By modeling conditional quantiles rather than the mean, this approach accommodates heterogeneity, non‐normal errors, and potential outliers common in cross‐country panel data, and it reveals whether improvements in the rule of law yield stronger gains in electoral integrity at specific points (e.g., bottom or top deciles) that would be obscured by conventional fixed‐ or random‐effects models | |
| Second, he talks about a dynamic panel. We don't see his results in the document, even though the problem of endogeneity (reverse causality) is obvious. | Thank you for this suggestion.  Panel Quantile Regression with individual fixed effects purges time‐invariant unobserved heterogeneity that could correlate with both the rule of law and electoral integrity, thereby mitigating omitted‐variable endogeneity across each conditional quantile.  By also including the one‐period lag of electoral integrity in our dynamic specification, we capture feedback effects and attenuate reverse‐causality bias without resorting to instrument‐based estimators | |
| Third, we also don't address the endogeneity problem related to omission bias. | Thank you for your comment on this issue. The study used Hausman test for this with the result affirming no issue with omiited variable | |
| Fourth, we have tables that look like software outputs. This is not elaborate. | Thanks for this suggestion.  We have modified the tables we obtained from the software, all redundancy have been remove and the current table only focuses on important details such as the coefficients and the significant levels. | |
| **Reviewer #3:** Thank you for the opportunity to review this manuscript.  The paper addresses an important and timely topic by exploring potential factors contributing to free and fair elections—specifically, the rule of law, electoral pluralism, and political rights.  Against the backdrop of limited democratization in many African countries despite regular elections, the authors seek to understand better which factors help foster elections that genuinely support democratic development. | Thanks for your comments and understanding of the study's direction. | |
| Methodological presentation and interpretation of results would benefit from restructuring to improve overall clarity.  I recommend that the authors be allowed to revise and resubmit the paper.  Currently, the empirical section is difficult to follow, particularly for readers who may not be familiar with advanced econometric techniques such as panel quantile regression.  Although the models are introduced sequentially, how they relate to one another is not always clear, nor is it clear why specific methodological choices—such as using quantile regression over other approaches—were made.  I was also unsure why panel OLS regression appears as a robustness check when earlier models using OLS with one- and two-way fixed effects are presented as main models.  Clarifying which models are considered the primary analyses and which serve as robustness checks and explaining the rationale behind these choices would help strengthen the empirical contribution. | Thank you for this suggestion.  We have revised and improved the presentation and enhanced the methodological flow.  We have thoroughly revised the manuscript  We have thoroughly improved the empirical section and improved its interpretation specifically the panel quantile regression  We have revised and detailed properly the reasons for selecting the techniques used  The study only used the panel OLS as a robustness check and the results validates the panel quantile regression.  Lines 405 – 410 have been added to clarify that, the study used the panel quantile regression as the primary analysis and the panel OLS is used as a robustness check. Employing both methods complements the analytical strength of each. Quantile regression is more flexible and insightful regarding distributional variations, while panel OLS regression provides a benchmark check for the consistency and reliability of the main findings. This dual approach strengthens the rigor of the research, increasing confidence in the conclusions drawn. | |
| Additionally, more details regarding the group referred to as the "twenty top democratizing countries" in Africa are needed**.** How were these countries selected?  Which data source (e.g., V-Dem?) was used to define this group?  **Why was the sample restricted to 20 countries?** Providing this information would help readers better understand the case selection and its relevance to the broader argument. Minor comments: | Thank you for your comment.  The explanation below have been added from lines 258 to 267  **The study used a panel data set of 20 African countries to examine the association between Free and Fair Elections and the Rule of Law.**  **According to Gerring (2007), using non-random selection procedures that focus on the most similar cases when analyzing cases is advantageous for gaining deeper insight. In this article, these are Africa's most democratizing countries, providing valuable insight into the factors that promote electoral integrity.**  **The top 20 democratizers represent a subset of African countries that have held regular elections and achieved a certain level of integrity that contributes to democratic improvement. Therefore, their selection is justified by their performance.**  **These 20 countries constitute approximately 40 per cent of all African nations, offering a meaningful and representative sample.** | |
| • The manuscript would benefit **from careful proofreading and editing for grammar, clarity, and consistency of voice (e.g., switching between “we” and “I”).** | Thank you for your comment.  The article has been proofread and edited for grammar and clarity by a professional editing service.  The authors preferred to use the third person to maintain objectivity and formality.  Hence, rather than We or I, it has been addressed as “ the current study” consistently across the study. | |
| • **There is some repetition in the discussion of data sources, particularly across Sections 3.1 and 3.2, which can make the argument harder to follow**. | Thank you for your great observation  The two sections have been rewritten as follows.  **3.1 Case Selection**  **Assuming all countries now practice a multiparty system and a focus on electoral plurality and political rights, we used a panel data set to examine** twenty top democratizing countries in Africa – Nigeria, Ghana, South Africa, Egypt, Kenya, Benin, Botswana, Cape Verde, Cote d'Ivoire, Gambia, Lesotho, Liberia, Madagascar, Malawi, Mauritius, Namibia, Senegal, Tanzania, Uganda and Zambia. **According to Gerring (2007), it is advantageous to use non-random selection procedures that focus on the most similar cases when analyzing cases to gain deeper insight. In this article, these are Africa's most democratizing countries, providing valuable insight into the factors that promote electoral integrity. The top 20 democratizers represent a subset of African countries that have held regular elections and achieved a certain level of integrity that contributes to democratic improvement. Therefore, their selection is justified by their performance. These 20 countries constitute approximately 40 per cent of all African nations, offering a meaningful and representative sample.**  **3.2 Variable and data source**  As a result of limited research on the impact of the rule of law, electoral pluralism, and political rights on electoral integrity, this current study tends to progress the research by **examining the association between Free and Fair Elections and the Rule of Law.** In addition, to resolve the limitation of previous studies that have omitted longitudinal impact on independent variables on free and fair elections, Autoregressive Distributed Lag (ARDL) that provides analysis on short-run and longrun relationships among variables regardless of stationary issues has been employed to examine the long-run effect. Times series data stretching for 17 years (2006-2023) have been collected from V-Dem (2024) for the rule of law, free and fair election, and the Economist Intelligence Unit for electoral pluralism. This study stands on the work of Edgeli (2018), investigating the global test of the democratization by-election hypothesis, | |
| • Ensure that all cited works appear in the reference list—for example, **Edgeli (2018) is mentioned in the text but not included in the references.** | Thank you for your comment.  All works cited have been included in the referecnes.  Edgeli 2018 was actually the below and has been corrected to Edgell et al., and added to refwerence list  Edgell, A. B., Mechkova, V., Altman, D., Bernhard, M., & Lindberg, S. I. (2018). When and where do elections matter? A global test of the democratization by elections hypothesis, 1900–2010. *Democratization*, *25*(3), 422-444. | |
|  |  | |
|  |  | |
