# Supplementary material for: Analysis of factors impacting electoral integrity in Africa between 2006–2023 - Examining the association between free and fair election and rule of law
Source: PLoS One. 2025 Oct 9;20(10):e0334505. doi: 10.1371/journal.pone.0334505 (PMC12510509; doi:10.1371/journal.pone.0334505)
Supplement: Attachment — Submitted filename: Response to Reviewer 2.docx [file pone.0334505.s004.docx]

**Dear Prof/Editor/Reviewers,**

We thank you and the reviewers for the valuable feedback provided on our manuscript. We have carefully revised the paper in line with the comments and suggestions received. Below, we provide a point-by-point response:

**Editorial Comments**

Comment 1:

We noticed that a statement in the conclusion section may be unclear or unsupported. Specifically, the statement “ROL consistently enhances electoral integrity across all African countries' contexts, indicating its foundational role in ensuring fair elections.” We believe this may be an overstatement and would recommend rephrasing to: “This suggests that ROL enhances electoral integrity across all African countries' contexts, indicating its foundational role in ensuring fair elections.”

**Response:**

**Thank you for pointing this out. We agree that the original wording could be interpreted as an overstatement. We have rephrased the statement in the conclusion as suggested to avoid overgeneralization.**

Comment 2:

Please review your reference list to ensure that it is complete and correct. If you have cited papers that have been retracted, please include the rationale for doing so in the manuscript text, or remove these references and replace them with relevant current references. Any changes to the reference list should be mentioned in the rebuttal letter.

**Response:**

**We have carefully reviewed the reference list. No retracted papers were cited, and no changes, removals, or replacements were required.**

**Reviewer Comments needing response**

Reviewer #1

Is the manuscript presented in an intelligible fashion and written in standard English? – Yes.

Additional comments – None.

Reviewer #3

Is the manuscript presented in an intelligible fashion and written in standard English? – No.

Comments: The reviewer recommends that the introduction should more clearly articulate the research question and the main aim of the paper earlier on. The reviewer also notes that grammar and spelling errors should be corrected throughout for clarity and readability.

**Response:**

We are grateful for these comments. The manuscript has been thoroughly proofread by an English editor with expertise in Political Science and International Relations. This ensured that all grammatical and typographical errors were corrected while maintaining contextual meaning. The tracked changes document highlights these edits.

Regarding the introduction, we have revised it to clearly articulate the research question and study aim earlier in the text. Specifically:

**General Research Question (Revised Introduction Section):**

Regular elections in African authoritarian countries have triggered debates over the conditions that contribute to electoral integrity. Elections were held in both the colonial and post-colonial eras, but in the colonial era, they were limited to a few legislative seats under colonial governance. In contrast, post-colonial elections expanded democratization by including parliamentary and presidential offices. Despite this expansion, the integrity of elections was increasingly questioned as autocratic regimes emerged, manipulating electoral rules, office tenures, and results to consolidate power. Hence, what are the systemic factors that contribute to free and fair elections?

**Specific Research Question (Revised Introduction Section):**

Building on this context, this study specifically examines whether the rule of law, electoral pluralism, and political rights significantly impact electoral integrity across African countries.

**Aim and Significance of the Study (Revised Introduction Section):**

The aim of this research is to shed light on the systemic factors that bolster or undermine electoral integrity by analyzing the relationship between free and fair elections and the rule of law. The significance of this study lies in its contribution to ongoing debates on credible elections in African democracies and its relevance for policymakers, election observers, and civil society organizations seeking to enhance transparency and accountability. Using panel data spanning 17 years and applying Panel Quantile Regression, the study provides nuanced insights into how the rule of law influences electoral integrity across the distribution of integrity scores—highlighting patterns that conventional methods may obscure.

These revisions directly address the reviewer’s comments and ensure that the research question, aim, and significance are clear and introduced earlier in the manuscript.

Also, the entire study have been examiend to ensure that all grammartical issues have been addressed.

**Summary of Changes**

Rephrased the conclusion statement to avoid overgeneralization (as suggested by the Editor).

Carefully reviewed references; no retracted or replaced sources.

Proofread the entire manuscript for grammar and clarity with assistance from an English editor.

Revised the introduction to present the research question, aim, and significance more explicitly and earlier in the manuscript.

We trust these revisions address the concerns of both the editorial team and the reviewers. Thank you for your thoughtful feedback and for the opportunity to improve our manuscript.

Sincerely,

Michael Oluchi Ikoko

Corresponding Author
